# Truth unveiled by time and the marbled definition of D2T-RA: retrospective analysis on the persistence of the difficult-to-treat status among refractory RA patients

**DOI:** 10.1186/s13075-024-03390-x

**Published:** 2024-09-17

**Authors:** Gilberto Cincinelli, Gabriella Maioli, Cristina Posio, Ennio Giulio Favalli, Francesca Ingegnoli, Roberto Caporali

**Affiliations:** 1 Department of Rheumatology and Medical Sciences, ASST Gaetano Pini-CTO, Milan, Italy; 2 Department of Clinical Sciences and Community Health, University of Milan, Milan, Italy

**Keywords:** Rheumatoid arthritis, Refractory disease, Difficult-to-treat, Persistent difficult-to-treat, b/tsDMARDs

## Abstract

**Background:**

The current EULAR definition of difficult-to-treat rheumatoid arthritis (D2T-RA) identifies patients with active disease refractory to multiple treatments at a single time point, without considering the persistence of this condition over time. The study aimed to assess difficult-to-treat rheumatoid arthritis (D2T-RA) over 12 months, considering persistence over time rather than a single time point, in a real-life cohort.

**Methods:**

In a single-center real-life cohort, demographic and clinic data were cross-sectionally collected for each patient at baseline and retrospectively over the previous 12 months bimonthly. For each timepoint, the prevalence of D2T-RA patients was calculated, and patients meeting the EULAR definition for at least 6 months were defined as persistent D2T-RA (pD2T-RA). Finally, the clinical characteristics associated with the time-based definition of pD2T-RA were analyzed.

**Results:**

Among 610 adult RA patients, 104 were refractory to ≥ 2 treatments. Initially, 41.3% met D2T-RA criteria, but only 27.9% fulfilled persistent D2T-RA (pD2T-RA) criteria over 6 months. The pD2T-RA group was associated with male gender, higher HAQ and Charlson Comorbidity Index scores, more failed treatments, and use of non-NSAID analgesics. Logistic regression linked pD2T-RA to higher SDAI and CRP values, and the use of glucocorticoids or analgesics. Chronic use of glucocorticoids was strongly associated with pD2T-RA.

**Conclusions:**

The application of a temporal criterion allowed for the selection of a subgroup of pD2T-RA patients who differ from those who meet the definition of D2T-RA only episodically. Chronic use of glucocorticoids was the factor most strongly associated with pD2T-RA status.

## Key messages

*What is already known about this subject?*

Since the institution of the D2T-RA definition, many efforts have been made to characterize this subpopulation's prevalence as well as clinical and demographical features. However, no studies have so far faced the temporal maintenance of the D2T-RA status.

*What does this study add?*

A definition of persistent or episodic D2T-RA is proposed according to the temporal trajectory of D2T-RA status, resulting in approximately 27% of D2T-RA patients persistently fulfilling the pD2T-RA definition. At each single time point, one-third of D2T-RA patients are represented by episodic D2T-RA patients.

*How might this impact on clinical practice?*

Persistent D2T-RA definition may serve to further homogenize the D2T-RA population.

## Introduction

The treat-to-target (T2T) strategy imposes the fulfillment of therapeutic targets in the management of rheumatoid arthritis (RA) [1], as highlighted by the European Alliance of Associations for Rheumatology (EULAR) recommendations on RA management [2]. Notably, despite advances in the field of targeted therapies, a sizeable proportion of RA patients still suffer the necessity to switch or swap between different biologic or targeted synthetic disease-modifying anti-rheumatic drugs (b/tsDMARDs) to achieve appropriate disease control.

More recently an EULAR experts task force introduced the notion of difficult-to-treat (D2T) RA patients [3], to dampen heterogeneities for research purposes. By this concept, criteria to be altogether fulfilled are reliant upon 1) the failure of at least 2 b/tsDMARDs with different mechanisms of action; 2) the presence of signs and/or symptoms of active disease; 3) the perception of a complex disease condition by either the physician or the patient.

While this definition has a key role in identifying more severe RA, it encompasses a heterogeneous group of patients, as shown by real-world data [4]. These criteria provide a glimpse of D2T-RA patients in a single moment in time, while an enhanced understanding of the duration (episodic or persistent nature) of this condition might facilitate a more discerning appraisal of the patients’ characteristics and therapeutic intervention.

Thus, the study objectives were four-fold. We first evaluated the prevalence of D2T-RApatients from a single-center cohort of RA patients. We then examined retrospectively how long D2T-RA patients maintained this condition over 12 months. Patients were arbitrarily classified as episodic D2T-RA (eD2T-RA: duration < 6 months) and persistent D2T-RA (pD2T-RA: duration ≥ 6 months). Last, we characterized e- and p-D2T-RA patients with clinical and laboratory parameters. Moreover, we assessed the rate of D2T-RA criteria fulfillment to identify the main driver(s) of the condition.

## Materials and methods

### Study design and patient selection

**Fig. 1:**
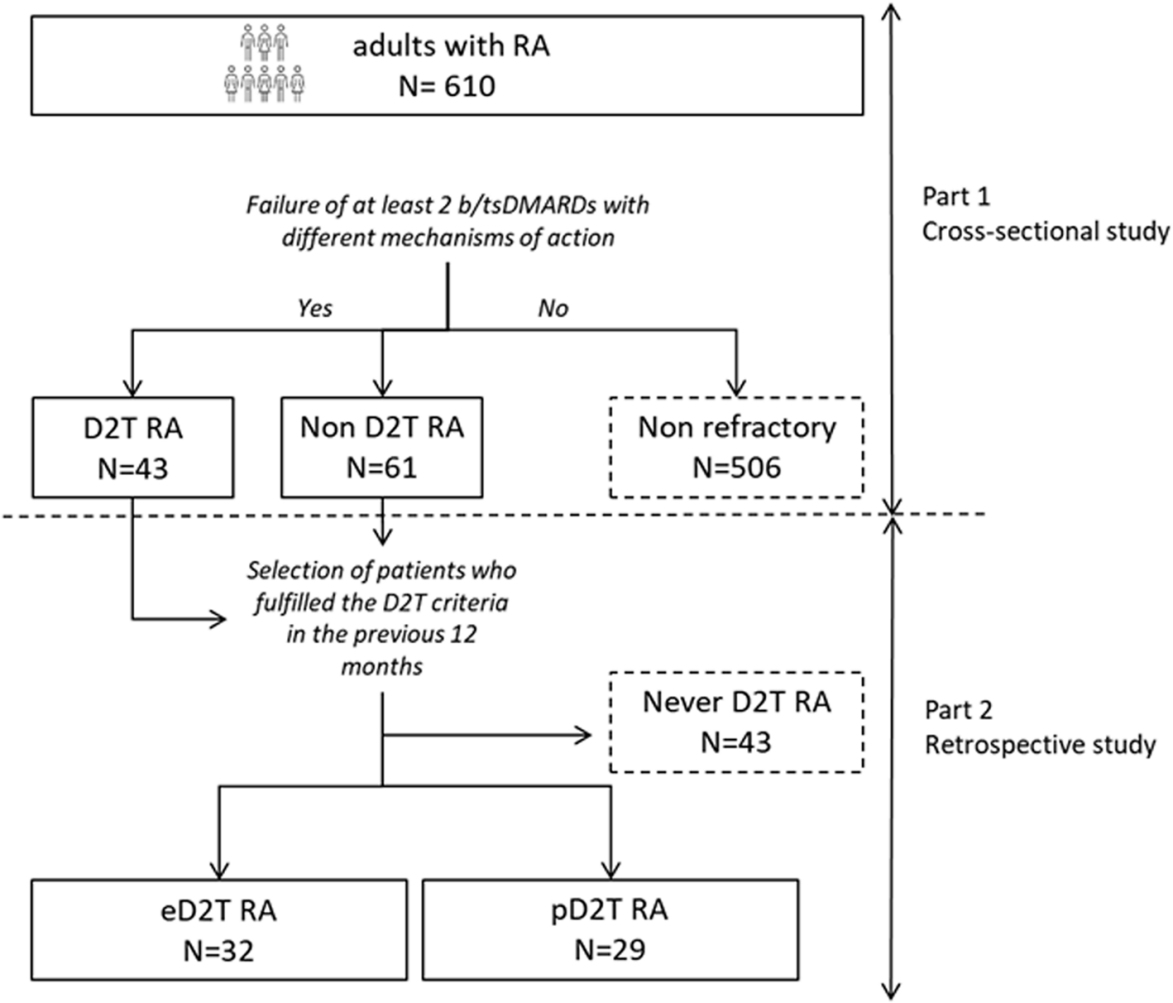
Study design. Abbreviations. RA: rheumatoid arthritis; DMARD: disease-modifying anti-rheumatic drug; bDMARD: biological DMARD; tsDMARD: targeted synthetic DMARD; D2T-RA: difficult-to-treat; e-D2T-RA: episodic D2T-RA; p-D2T-RA: persistent D2T-RA

Our study included a real-life cohort of adult RA patients [5] treated with b/tsDMARDs at our dedicated outpatient clinic in a tertiary care center in Milan, Italy. According to our clinical practice, RA patients, who signed the informed consent for any analysis of their clinical data, are recorded in a longitudinal observational registry (Ethics Committee 138_1999). In conformity with Lombardy legislation, patients treated with oral or subcutaneous b/tsDMARDs receive a drug supply every two months during visits, while visits and infusions of patients treated with intravenous therapies are scheduled based on the mechanisms of action (MoA) of bDMARD. As shown in Fig. 1, the study encompasses two different parts: a cross-sectional and a retrospective analysis.

### Part 1—cross-sectional analysis

We included consecutive, adult RA patients from October 2022 through April 2023. At the time of the visit (*t0*), we collected demographical (gender, age, smoking habit, body mass index), clinical (disease duration, seropositivity for rheumatoid factor (RF) and/or anti-citrullinated antibodies (ACPAs), presence of erosive disease, presence of comorbidities either singularly and collected as Charlson Comorbidity Index to reflect the burden of comorbid disease [6], clinimetric data (number of tender and swollen joints on 28 sites (TJC and SJC, respectively), patient and physician global assessment on a 0 to 10 scale (PGA and PhGA, respectively), Numeric Pain Rating Scale (NRS 0–10), Simplified Disease Activity Index (SDAI) [7], laboratory data (C-Reactive Protein (CRP) values), and data regarding ongoing and previous therapies namely number of previous b/tsDMARDs, number of previous b/tsDMARDs MoA, the time elapsed between diagnosis and the first b/tsDMARD (time-to-biologic) and between the diagnosis and the failure of the first 2 MoAs (time-to-refractory), current and previous conventional synthetic DMARDs (csDMARDs), current glucocorticoid use and dosage, current analgesic and non-steroidal anti-inflammatory drugs (NSAIDs).

According to the EULAR definition [3], we identified the proportion of D2T-RA patients from our cohort at *t0* as 1) patients who failed at least 2 b/tsDMARDs mechanisms of action after failing a previous csDMARD; 2) signs suggestive of active or progressive disease, defined as either i) at least moderate disease activity according to SDAI or CDAI score, or ii) signs (including acute phase reactants) and/or symptoms suggestive of active disease, or iii) inability to taper glucocorticoid therapy below 7.5 mg/day prednisone or equivalent, or iv) rapid radiographic progression, or v) symptoms reducing the quality of life, despite well-controlled disease according to standards, or 3) perception of problematic management of signs and symptoms by either the physician or patient.

### Part 2—Retrospective data analysis

RA patients, included in part 1 analysis, who failed at least 2 b/tsDMARDs MoA ( refractory) were selected for a retrospective study including data from the previous 12 months (Fig. 1). In the retrospective analysis, patients were classified as difficult to treat according to the criteria of the EULAR D2T-RA definition, as described previously.

For each time point (*t0*, then retrospectively every two months from *t-2* to *t-12*), we identified D2T-RA patients according to the EULAR criteria [3], and we arbitrarily classified patients as episodic D2T-RA (eD2T-RA) and persistent D2T-RA (pD2T-RA) according to the duration of the D2T-RA state for less or more than a total of 6 months (i.e. 50% of the observation period), respectively. For the identification of symptoms of impaired quality of life from the retrospective data collection from clinical records, we employed the NRS score for pain as a proxy for criteria 2.v.

A comparison between pD2T-RA and eD2T-RA patients was carried out both with univariate analysis and multivariate analysis using logistic regression analysis.

Furthermore, we assessed the contribution of three objective criteria of the D2T-RA definition in the whole refractory population (i.e. at least moderate disease activity according to SDAI; a raised CRP value with no explanation other than disease activity; inability to taper glucocorticoid dosage) at t0, t-6 and, t-12, as well as by calculating the proportion of each criteria fulfillment during the observation period in the pD2T-RA and eD2T-RA subgroups as follows: (number of events of criteria fulfillment per patient/total observations per patient) × 100.

### Statistical analysis

All statistical analyses were performed with IBM SPSS Statistics for Windows, Version 28.0. Armonk, NY: IBM Corp.

Categorical variables were depicted as absolute numbers and percentages. The normal distribution of continuous variables was assessed with the Kolmogorov–Smirnov test. Continuous variables with normal distribution were described as means ± standard deviations, while non-normally distributed continuous variables with median values and interquartile ranges. Then, we performed univariable analysis to evaluate any differences in terms of demographic and clinical features between pD2T-RA patients and e-D2T-RA patients. Univariable analysis between categorical variables was performed with chi-squared tests, and with t-student test for normally distributed continuous variables. Kruskal–Wallis test was performed as a non-parametric test to confront median values between groups. Multivariate analyses were conducted using logistic regression analysis including independent variables of interest from the results of the univariate analysis. We then calculated the concordance of D2T-RA patients across 3 time points (*t0*, *t-6,* and *t-12*) to explore the consistency of the D2T-RA for single patients over time and to obtain a quantitative measurement of such persistency. Intra-patients’ persistency was calculated both as concordance rates (i.e. patients maintaining the D2T-RA or non-D2T-RA status at two different time points) and as Cohen κ coefficients.

Lastly, we calculated the proportion of each criteria fulfillment during the observation period subgroups as follows: (number of events of criteria fulfillment per patient/total observations per patient) × 100 and confronted the median values between pD2T-RA and eD2T-RA patients performing the Kruskal–Wallis test. We also performed an ANOVA analysis between the fulfillment rates of criteria within the pD2T-RA population (variables distributed continuously within this population) to unveil any differences between rates, possibly followed by a Tukey test whether any significance would emerge.

## Results

### Patients’ demographics at baseline

We enrolled 610 adult RA patients, among which 507 (83.1%) were female. At baseline, the mean age was 58.1 ± 12.5 years, and the mean disease duration of 17.4 ± 11.7 years. 115 patients (19%) were current smokers, and the median BMI was 23 (IQR 21 – 26). As to the disease characteristics, 66.6% were seropositive for RF and/or ACPAs, 9% presented with a previous diagnosis of fibromyalgia, and 48.5% suffered from symptomatic osteoarthritis (OA). 311 patients (50.9%) were receiving their first b/tsDMARDs, while 104 (17.05%) had previously failed at least 2 b/tsDMARDs with different mechanisms of action (i.e., refractory patients). Approximately half of the patients (49.7%) were receiving b/tsDMARDs without concomitant csDMARDs (monotherapy), 214 patients (35.1%) were currently prescribed systemic corticosteroid therapy, and among these 138 (22.6%) at a dose lower than 7.5 mg of prednisone equivalent/die. Table 1 summarizes the demographics and baseline characteristics of our cohort.

**Table 1:** Baseline clinical and demographic characteristics of patients with rheumatoid arthritis were included in the cross-sectional analysis

	**RA cohort treated with b/tsDMARDs (*N* = 610)**
**Demographic**	
Age (years) mean ± SD	58.1 ± 12.5
Female, (n, %)	507 (83.1%)
BMI (median, IQR)	23 (21 – 26)
Current Smokers (n, %)	115 (18.9%)
**Disease characteristics**	
RF and/or ACPA positive, (n, %)	387 (66.6%)
Disease duration (years) mean ± SD	17.4 ± 11.7
PGA 0–10 (median, IQR)	3 (1 – 5)
NRS 0–10 (median, IQR)	3 (0 – 5)
PhGA 0 -10 (median, IQR)	0 (0 – 1)
CRP (mg/dL) (median, IQR)	0.18 (0.07 – 0.5)
SDAI mean ± SD	8.2 ± 7.1
HAQ (median, IQR)	0.375 (0.125 – 0.75)
Time-to-biologic (months) mean ± SD	98.1 ± 91.9
Time-to-refractory (months) mean ± SD	77.3 ± 54.1
**Selected comorbidities**	
Osteoarthritis (n, %)	296 (48.5%)
Fibromyalgia (n, %)	55 (9%)
CCI (median, IQR)	2 (1 – 3)
**Current therapy**	
Mechanism of action, n (%)	
TNFα inhibitors	291 (47.7%)
IL-6 inhibitors	90 (14.8%)
Anti-CD20	40 (6.6%)
Anti-CTLA4	80 (13.1%)
JAK inhibitors	108 (17.7%)
IL-1 inhibitors	1 (0.1%)
b/tsDMARDs line (median, IQR)	1 (1 – 2)
csDMARDs (n, %)	307 (50.3%)
Glucocorticoids (n, %)	214 (35.1%)
Low dose glucocorticoids (n, %)	138 (22.6%)
NSAIDs (n, %)	171 (28%)
Analgesic use (n,%)	90 (14.8%)

*DMARD* Disease-modifying anti-rheumatic drug, *bDMARD* Biological DMARD, *tsDMARD* Targeted synthetic DMARD, *csDMARD* Conventional synthetic DMARD, *RF* Rheumatoid factor, *ACPA* Anti-citrullinated protein antibody, *BMI* Body mass index, *PGA* Patient global assessment, *NRS* Numerical rating scale, *PhGA* Physician global assessment, *HAQ* Health assessment questionnaire, *CRP* C-reactive protein, *CCI* Charlson comorbidity index, *TNF* Tumor necrosis factor, *IL-6* Interleukin 6, *CTLA4* Cytotoxic T-lymphocytes antigen 4, *JAK* Janus kinase, *IL-1* Interleukin 1, *NSAID* Non-steroidal anti-inflammatory drug

### Identification of persistent and episodic D2T-RA patients

At *t0*, 43/104 refractory RA patients (41.3%) fulfilled the D2T-RA criteria. Compared to non-D2T-RA patients, D2T-RA patients were less frequently female (80.95% vs 95.16%; *p* = 0.048), were younger (58.5 ± 11.4 years vs 62.7 ± 10.2; *p* = o.049), and yet had a longer disease duration (24.5 ± 11.58 years vs 18 ± 12.41 years; *p* = 0.049). Moreover, D2T-RA patients showed higher rates of ongoing corticosteroid therapy (81.4% vs 34.4%; *p* < 0.001), higher CRP values [0.66 (0.16 – 1.59) mg/dL vs 0.2 (0.06 – 0.4) mg/dL; *p* = 0.009], higher TJC-28 [2 (0 -6) vs 0 (0 – 0); *p* < 0.001] and SJC-28 [1 (0 – 3) vs 0 (0 -0); *p* < 0.001], higher PGA and NRS0-10 [6 (4 – 7) vs 3 (1.5 – 5); *p* < 0.001; and 6 (4.25 – 7) vs 3 (0 – 5); *p* = 0.005, respectively], higher PhGA [2 (0 – 3) vs 0 (0 – 0); *p* < 0.001], higher SDAI scores (13.8 ± 7.5 vs 4.3 ± 2.8; *p* < 0.001), and higher HAQ scores [0.75 (0.25 – 1.25) vs 0.375 (0.125 – 0.75); *p* = 0.027]. No differences between time-to-biologic and time-to-refractory, as well as between other demographic and clinical characteristics emerged, as described in Table 2.

**Table 2:** Characteristics of RA refractory to at least 2 b/tsDMARDs with different mechanisms of action classified as D2T-RA or non-D2T-RA at baseline (*t0*)

	**RA refractory to at least 2 b/tsDMARDs with different mechanisms of action**		
	**Non D2T-RA at *t0*** **(*N* = 61)**	**D2T-RA at *t0*** **(*N* = 43)**	***p*-values**
**Demographic**			
Age (years) mean ± SD	62.7 ± 10.2	58.5 ± 11.4	**0.049**
Female sex (n, %)	58 (95.1%)	35 (81.4%)	**0.048**
BMI (median, IQR)	23 (20.5 – 26)	23.5 (22 – 27.5)	0.59
Smokers (n, %)	15 (24.6%)	7 (16.3%)	0.57
**Disease characteristics**			
RF and/or ACPA positive (n, %)	38 (62.3%)	28 (65.1%)	0.96
Disease duration (years) mean ± SD	19.5 ± 10.4	23.8 ± 11.4	**0.049**
PGA 0–10 (median, IQR)	3 (1.5 – 5)	6 (4 – 7)	**< 0.001**
NRS 0–10 (median, IQR)	3 (0 – 5)	6 (4.25 – 7)	**0.005**
PhGA 0 -10 (median, IQR)	0 (0 – 0)	2 (0 – 3)	**< 0.0001**
CRP (mg/dL) (median, IQR)	0.2 (0.06 – 0.4)	0.66 (0.16 – 1.59)	**0.009**
SDAI (mean ± SD)	4.3 ± 2.8	13.8 ± 7.5	**< 0.001**
HAQ (median, IQR)	0.375 (0.125 – 0.75)	0.75 (0.25 – 1.25)	**0.027**
Time-to-biologic (months) mean ± SD	98.1 ± 91.9	122.4 ± 106.1	0.15
Time-to-refractory (months) mean ± SD	77.3 ± 54.1	74.6 ± 61.2	0.83
**Selected comorbidities**			
Osteoarthritis (n, %)	39 (63.9%)	29 (67.4%)	0.71
Fibromyalgia (n, %)	8 (13.1%)	9 (20.9%)	0.29
CCI (median, IQR)	2 (1 – 3.5)	2 (1 – 2)	0.45
**Current therapy**			
Mechanism of action, n (%)			
TNFα inhibitors	11 (18.1%)	8 (18.6%)	0.601
IL-6 inhibitors	15 (24.6%)	4 (9.3%)	0.07
Anti-CD20	10 (16.4%)	7 (16.3%)	0.988
Anti-CTLA4	6 (9.8%)	6 (14%)	0.517
JAK inhibitors	19 (31.1%)	17 (39.5%)	0.376
IL-1 inhibitors	0 (0%)	1 (2.3%)	0.413
b/tsDMARDs line (median, IQR)	4 (3 – 5)	5 (4 – 6)	0.24
csDMARDs (n, %)	31 (50.8%)	24 (55.8%)	0.62
Glucocorticoids (n, %)	21 (34.4%)	35 (81.4%)	**< 0.001**
Low-dose glucocorticoids (n, %)	20 (32.8%)	8 (18.6%)	
NSAIDs (n, %)	17 (27.9%)	13 (30.2%)	0.74
Analgesic use (n, %)	13 (21.3%)	12 (27.9%)	0.44

*DMARD* Disease-modifying anti-rheumatic drug, *bDMARD* Biological DMARD, *tsDMARD* Targeted synthetic DMARD, *csDMARD* Conventional synthetic DMARD, *RF* Rheumatoid factor, *ACPA* Anti-citrullinated protein antibody, *BMI* Body mass index, *PGA* Patient global assessment, *NRS* Numerical rating scale, *PhGA* Physician global assessment, *HAQ* Health assessment questionnaire, *CRP* C-reactive protein, *CCI* Charlson comorbidity index, *TNF* Tumor necrosis factor, *IL-6* Interleukin 6, *CTLA4* Cytotoxic T-lymphocytes antigen 4, *JAK* Janus kinase, *IL-1* Interleukin 1, *NSAID* Non-steroidal anti-inflammatory drug

**Fig. 2:**
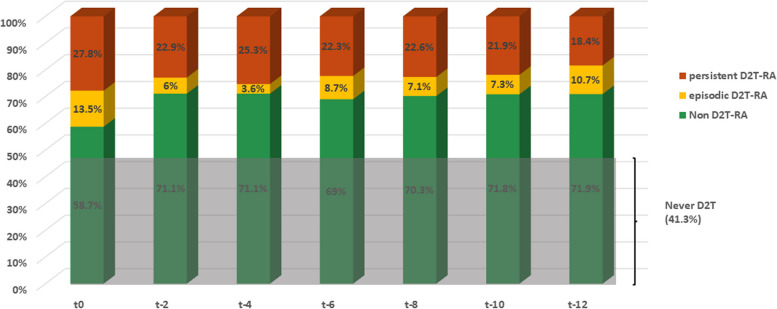
Prevalence of D2T-RA patients among refractory RA patients in the 12 months of observation. Prevalence of D2T-RA patients among refractory RA patients in the 12 months of observation. For each time point, the overall frequency (percentage) of pD2T-RA (red), eD2T-RA (yellow), and non-D2T-RA (green) is reported. Noteworthy, 41.3% of refractory patients were never D2T-RA (grey area at the bottom). Abbreviations. RA: Rheumatoid Arthritis; pD2T-RA: persistent difficult-to-treat; eD2T-RA: episodic difficult-to-treat; D2T-RA: difficult-to-treat

**Fig. 3:**
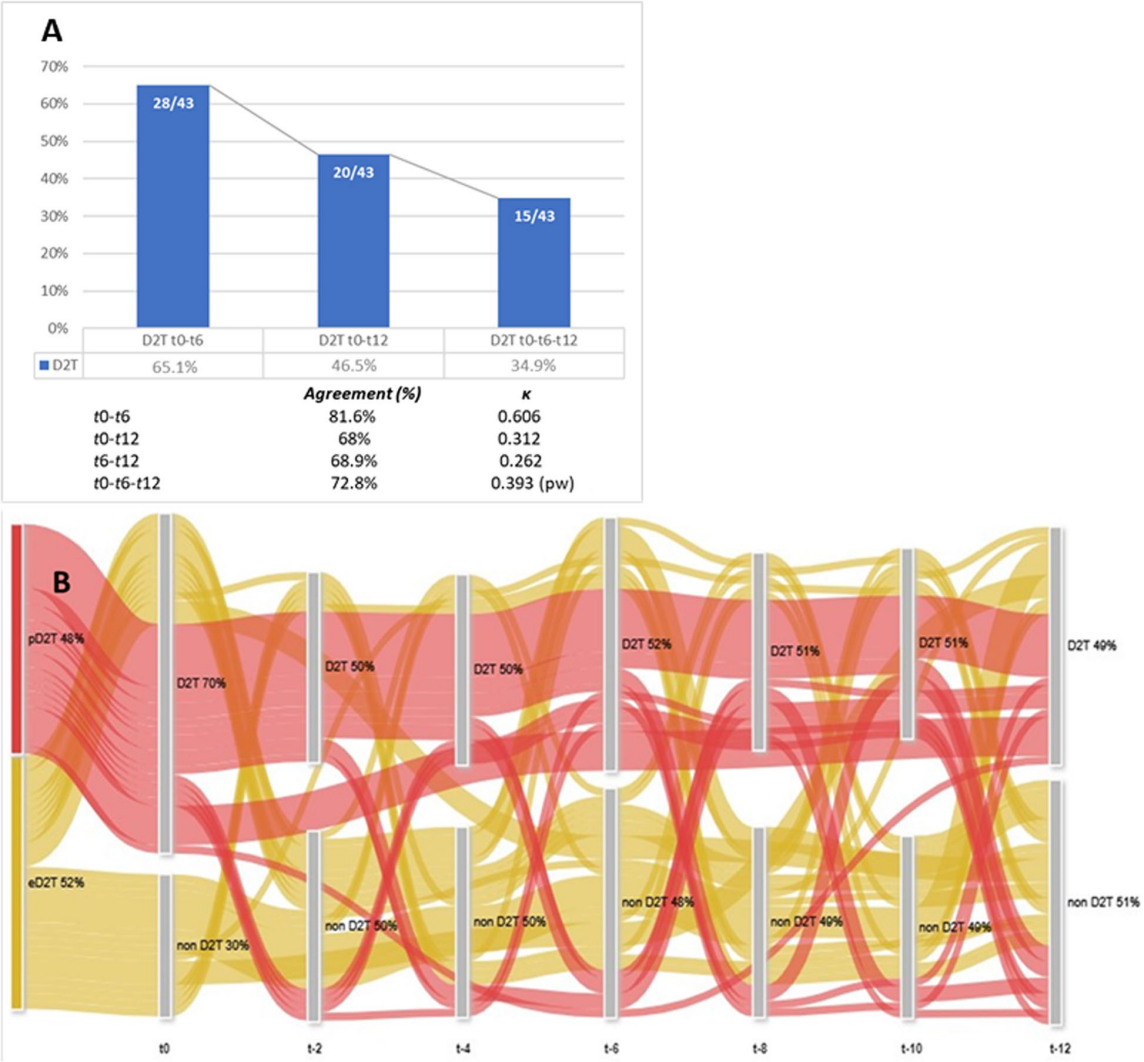
**A** Agreement and Cohen’s κ of D2T-RA definition. **B** Sankey diagram showing the fluctuations of pD2T-RA and eD2T-RA patients. **A** Analysis of agreement and Cohen’s κ of D2T-RA definition among patients at different time points during the 12 months of observation. **B** Sankey diagram showing the fluctuations of D2T-RA state of pD2T-RA (red links) and eD2T-RA patients (yellow links). Notably, the red links almost uniformly flow through the D2T-RA nodes from t0 to t-12 with few, scattered losses. On the other hand, the oscillations of yellow links between the D2T-RA and the non-D2T-RA status are more frequent and more conspicuous along the 12 months follow up (never D2T-RA patients were excluded from the analysis). Abbreviations. D2T-RA: difficult-to-treat rheumatoid arthritis; pw: pairwise; pD2T-RA: persistent difficult-to-treat; eD2T-RA: episodic difficult-to-treat

In our cohort, D2T-RA43/104 patients (41.3%) never fulfilled the D2T-RA definition criteria in the 12 months of observation. The prevalence of D2T-RA patients among the 104 refractory patients in the previous 12 months ranged constantly from 29 to 31% (Fig. 2). In detail, D2T-RA patients were 24/83 (28.9%) at *t-2* and *t-4*, 32/103 (30.8%) at *t-6*, 25/84 at *t-8* (29.8%), 24/82 (29.3%) at *t-10*, and 30/103 (28.8%) at *t-12*. Furthermore, among the 32 D2T-RA patients at *t-6*, 28/32 (87.5%) were D2T-RA both at *t0* and *t-6* (81.6% concordance, κ = 0.606), while among the 30 D2T-RA patients at *t-12*, 20/30 (66.7%) were D2T-RA at *t0* too (68% concordance, κ = 0.312) (Fig. 3A). Globally, 15/43 patients (34.9%) simultaneously fulfilled D2T-RA status at *t0*, *t-6*, and *t-12* (68% concordance, κ pairwise = 0,393). As depicted in the Sankey diagram in Fig. 3B, at a single-patient resolution approximately one-third of D2T-RA patients at each timepoint was represented by different eD2T-RA, likely explaining the low agreement score observed among D2T-RA patients.

As expected, during each time point the frequency of pD2T-RA among refractory patients was higher than eD2T-RA. In detail, the prevalence of pD2T-RA patients ranged from 18.4% at *t-12* (19 out of 103 patients) to 27.8% at *t0* (29 out of 104 patients) (Fig. 2).

### Comparison between pD2T-RA and eD2T-RA

The univariate analysis between pD2T-RA and eD2T-RA patients showed a higher frequency of male patients (14.1% vs 5.3%; *p* = 0.01), higher PGA and PhGA scores [6 (5 – 8) vs 3 (1 – 6); *p* < 0.001; and 2 (0 – 3) vs 0 (0 – 1); *p* = 0.002, respectively], higher NRS 0–10 [6 (5 – 7) vs 4 (1 – 6); *p* = 0.02], SDAI scores (14.7 ± 8.4 vs 5.7 ± 4.5; *p* < 0.001), and HAQ scores [1 (0.5 – 2.25) vs 0.375 (0.125 – 0.875); *p* = 0.031] as well as higher number of previously failed b/tsDMARDs [5 (4 – 6) vs 4 (3 – 4); *p* = 0.028] and use of non-NSAIDs analgesic therapy (37.9% vs 18.7%; *p* = 0.039) among pD2T-RA patients. We observed a trend for higher time-to-biologic in pD2T-RA, however not statistically significant (137 ± 112.9 months vs 95.8 ± 92.5 months; *p* = 0.057). No differences emerged in terms of disease duration, time-to-refractory, prevalence of RF and/or ACPA positivity, symptomatic osteoarthritis, and fibromyalgia (Table 3). Multivariate analyses applying logistic regression confirmed that SDAI scores (*p* < 0.001; OR 1.232, 95% CI 1.085–1.399), CRP values (*p* = 0.035; OR 2.217, 95% CI 1.059–4.645), use of glucocorticoid (*p* = 0.004; OR 24.29, 95% CI 2.756–214.085), and use of non-NSAIDS analgesics (*p* = 0.046; OR 4.502, 95% CI 1.024–19.786) were positively associated with the persistency of the D2T-RA status (Table 3).

**Table 3:** Characteristics of persistent and non-persistent D2T-RA patients

	**RA refractory to at least 2 b/tsDMARDs with different mechanisms of action**		**Univariate analysis**	**Multivariate analysis**	
	**Persistent D2T-RA (*n* = 29)**	**Episodic D2T-RA (*N* = 75)**	***p*-values**	**OR (95% CI)**	***p*-values**
**Demographic**					
Age (years) mean ± SD	60.0 ± 9.5	61.3 ± 11.3	0.593	0.98 (0.92–1.05)	0.62
Female sex (n, %)	22 (75.9%)	71 (94.7%)	**0.01**	3.20 (0.47–21.79)	0.23
BMI (median, IQR)	23.5 (23 – 27)	23 (21 – 26)	0.75	-	-
Smokers (n, %)	5 (17.2%)	17 (22.7%)	0.69	-	-
**Disease characteristics**					
RF and/or ACPA positive (n, %)	22 (75.9%)	44 (63.8%)	0.157	-	-
Disease duration (years) mean ± SD	25.1 ± 11.8	19.8 ± 10.4	**0.026**	1.04 (0.97–1.11)	0.315
PGA 0–10 (median, IQR)	6 (5 – 8)	3 (1 – 6)	**< 0.001**	-	-
NRS 0–10 (median, IQR)	6 (5 – 7)	4 (1 – 6)	**0.02**	-	-
PhGA 0 -10 (median, IQR)	2 (0 – 3)	0 (0 – 1)	**0.002**	-	-
CRP (mg/dL) (median, IQR)	1.02 (0.23 – 2.31)	0.2 (0.06 – 0.41)	**0.004**	**2.22 (1.06–4.65)**	**0.035**
SDAI (mean ± SD)	14.7 ± 8.4	5.7 ± 4.5	**< 0.001**	**1.23 (1.09–1.39)**	**0.001**
HAQ (median, IQR)	1 (0.5 – 2.25)	0.375 (0.125 – 0.875)	**0.031**	-	-
Time-to-biologic (months) mean ± SD	137 ± 112.9	95.8 ± 92.5	0.057	-	-
Time-to-refractory (months) mean ± SD	72.8 ± 62.8	77.4 ± 54.8	0.731	-	-
**Selected comorbidities**					
Osteoarthritis (n, %)	22 (75.9%)	46 (61.3%)	0.163	-	-
Fibromyalgia (n, %)	6 (20.7%)	11 (14.7%)	0.456	-	-
CCI (n, %)	2 (1 – 3)	2 (1 – 3)	0.446	-	-
**Current therapy**					
Mechanism of action, n (%)					
TNFα inhibitors	5 (17.2%)	14 (18.6%)	0.878		
IL-6 inhibitors	1 (3.5%)	18 (24%)	0.021		
Anti-CD20	5 (17.2%	12 (16%)	0.878		
Anti-CTLA4	4 (13.8%)	8 (10.7%)	0.735		
JAK inhibitors	13 (44.8%)	23 (30.7%)	0.173		
IL-1 inhibitors	1 (3.5%)	0 (0%)	0.279		
b/tsDMARDs line (median, IQR)	5 (4 – 6)	4 (3 – 5)	**0.028**	-	-
Current csDMARDs (n, %)	18 (62.1%)	37 (49.3%)	0.243	-	-
Glucocorticoids, (n, %)	25 (86.2%)	31 (41.3%)	**< 0.001**	**24.29 (2.76–214.09)**	**0.004**
Low-dose glucocorticoids, (n, %)	5 (17.2%)	23 (30.7%)			
NSAIDs (n, %)	7 (24.1%)	23 (31.1%)	0.485	-	-
Analgesic use (n, %)	11 (37.9%)	14 (18.7%)	**0.039**	**4.5 (1.02–19.79)**	**0.046**

*DMARD* Disease-modifying anti-rheumatic drug, *bDMARD* Diological DMARD, *tsDMARD* Targeted synthetic DMARD, *csDMARD* Conventional synthetic DMARD, *RF* Rheumatoid factor, *ACPA* Anti-citrullinated protein antibody, *BMI* Body mass index, *PGA* Patient global assessment, *NRS* Numerical rating scale, *PhGA* Physician global assessment, *HAQ* Health assessment questionnaire, *CRP* C-reactive protein, *CCI* Charlson comorbidity index, *TNF* Tumor necrosis factor, *IL-6* Interleukin 6, *CTLA4* Cytotoxic T-lymphocytes antigen 4, *JAK* Janus kinase, *IL-1* Interleukin 1, NSAID: non-steroidal anti-inflammatory drug

### Definition criteria contribution to the persistency of D2T-RA status

**Fig. 4:**
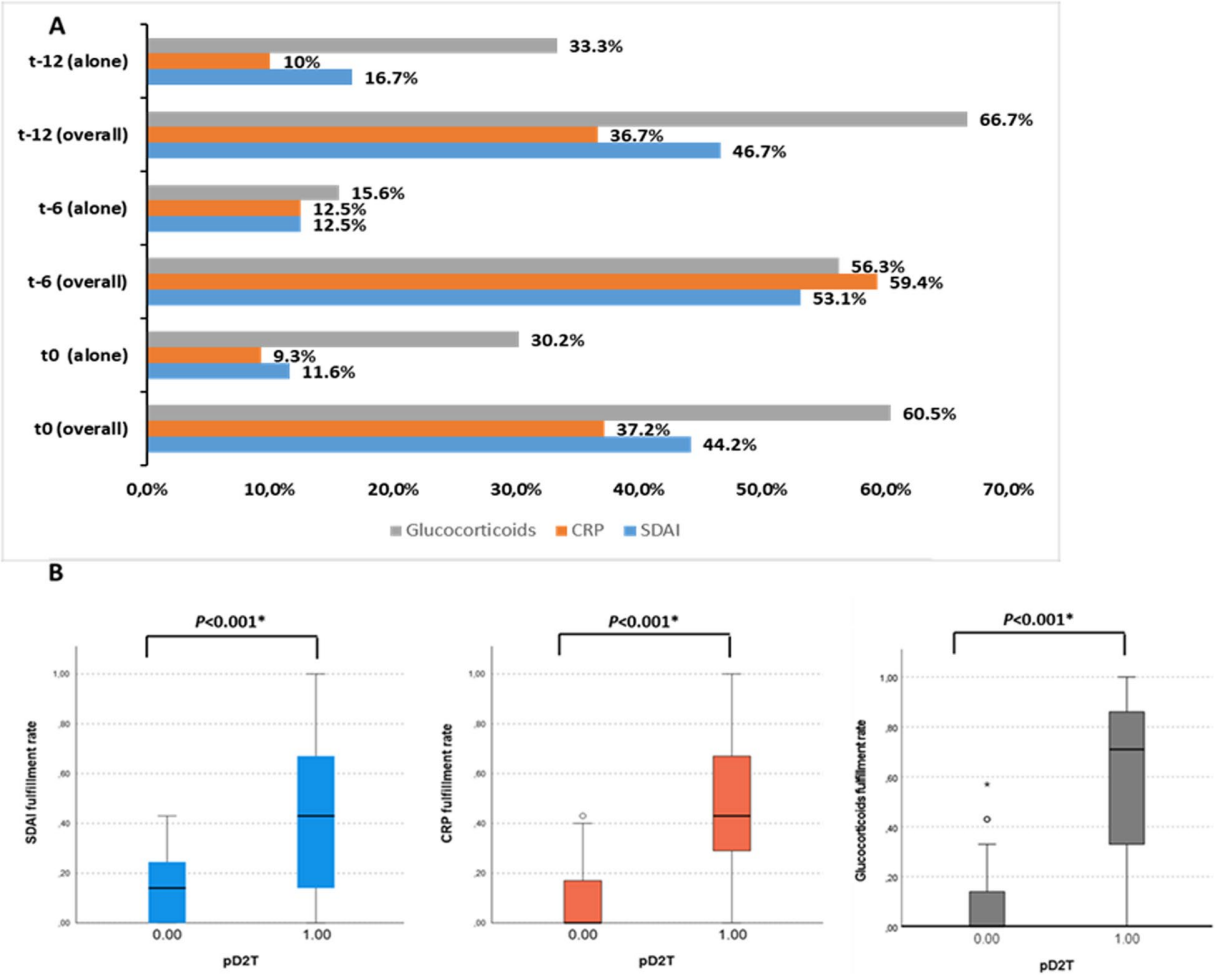
**A** Rate of criteria fulfillment at each timepoint and (**B**) between pD2T-RA and eD2T-RA. **A** Rate of criteria fulfillment at each timepoint, either including all events (overall) or the rate of definition fulfillment for a single criterion (alone). **B** Median rate of criteria fulfillment between pD2T-RA and eD2T-RA (excluding patients that never fulfilled the D2T-RA definition) along the 12-month observation period. Abbreviations. SDAI: Simplified Disease Activity Index; CRP: C-reactive protein; pD2T-RA: persistent difficult-to-treat

Among the 43/104 D2T-RA patients identified at t0, we found that 19/43 (44.2%) had a moderate/high disease activity according to the SDAI score, 16/43 (37.2%) presented with an otherwise unexplained raised CRP value, and 26/43 (60.5%) were treated with glucocorticoid at a dosage of at least 7.5 mg day of prednisone equivalent. Interestingly, 13/43 patients (30.2%) patients were defined as D2T-RA only for the fulfillment of the inability to taper glucocorticoid criterion, while only 11.6% and 9.3% fulfilled the moderate disease activity and the raised CRP criteria alone, respectively. At *t-6*, the moderate disease activity criterion was fulfilled by 17/32 D2T-RA patients (53.1%), the raised CRP criterion by 19/32 patients (59.4%), and the glucocorticoid criterion by 18/32 (56.3%). At *t-12*, the moderate disease activity raised CRP, and glucocorticoid criteria were fulfilled by 14/30 (46.7%), 11/30 (36.7%), and 20/30 (66.7%) D2T-RA patients, respectively. Like at *t0*, at t-12 10/30 (33.3%) patients were classified as D2T-RA only for the fulfillment of the glucocorticoid criteria, compared to 16.7% and 10% who fulfilled exclusively the moderate disease activity and raised CRP criteria, respectively (Fig. 4A).

The assessment of the rate of definition criteria fulfillment during the observation time (total of *N* = 642 observations) revealed higher median rates of moderate disease activity criterion fulfillment (0.43, IQR 0.54 vs 0.0, IQR 0; *p* < 0.001), higher rates of raised CRP values criterion fulfillment (0.43, IQR 0.47 vs 0.0, IQR 0; *p* < 0.001), and higher rates of glucocorticoid criteria fulfillment (0.71, IQR 0.6 vs 0.0, IQR 0.14; *p* < 0.001) in the pD2T-RA population compared to the eD2T-RA patients (Fig. 4B). Interestingly, no patients with rapid radiographic progression were identified, while all patients with a high NRS score were fulfilling the moderate disease activity criterion. The ANOVA analysis conducted between the mean criteria fulfillment rate in the pD2T-RA population revealed no significant differences between fulfillment rates (*p* = 0.053). For the ANOVA analysis was only slightly not significant, the Tukey test was conducted and showed a trend for a more frequent inability to taper glucocorticoids (*p* = 0.083 vs moderate disease activity; *p* = 0.093 vs CRP).

## Discussion

Our study provides both a cross-sectional and a longitudinal profiling of D2T-RA patients, affording insights into the clinical aspects underpinning this condition. To our knowledge, our study was the first to evaluate the trajectory of the D2T-RA status among RA patients and to weight of criteria fulfillment on the D2T-RA definition. The EULAR definition provides definite criteria to identify and categorize this complex population among RA cohorts [3, 8–11], with the principal aim to create a homogeneous population. These criteria were developed primarily for scientific purposes, and little is known about their utilization in routine clinical practice. This definition could have a potentially relevant value in clinical settings to help physicians' and patients' decision-making process in challenging cases [2]. However, the EULAR definition sets as mandatory criteria the failure of at least two b/tsDMARDs with different mechanisms of action. During the chronic disease history of each patient – as it is observed in clinical practice – RA evolves with variable clinical courses and it is subjected to different disease-related and non-disease-related circumstances that may affect disease activity and therapeutic strategies (i.e., aging, occurring comorbidities, infective diseases, pregnancy and breastfeeding, therapy-related adverse reactions, among the others). Nevertheless, the EULAR task force does not address any hierarchical difference among modalities of treatment failure (like primary or secondary inefficacy, adverse events) nor the time necessary to develop the refractory condition [11]. As it was hypothesized that such differences may underlie diverse biological substrates from which D2T-RA patients emerge [11, 12], no biological, serological, or genetic biomarkers capable to identify D2T-RA status are yet available at our disposal [13, 14]. We suggest that expanding the analysis of D2T-RA patients on a temporal dimension may further dissect such a complex population.

We applied this concept to our real-life cohort of RA to explore, for the first time, the time-related frame of the D2T-RA status. In particular, the term D2T-RA encompasses a broad range of conditions, and it is of paramount importance the evaluate time-dependent criteria to avoid the misreading of patients fulfilling the D2T-RA criteria only by temporary fluctuations of their disease activity state (for example in cases of temporary suspension of immunosuppressant therapies due to infections, certain vaccinations, or surgical procedures, circumstances that could befall for refractory patients as well as patients receiving their first b/tsDMARD).

Through the analysis of our population, we observed that the overall proportion of D2T-RA patients remained stable over time, with a frequency similar to that reported in other papers published so far [15–17]. However, concordance analysis demonstrated that only slightly more than one-third of D2T-RA patients observed at the time of enrollment persisted as D2T-RA in the previous 12 months. Further, approximately one-quarter of refractory patients fulfilled our definition of persistent D2T-RA. To our knowledge, this is the first report of longitudinal analysis provided on D2T-RA patients available. Our analysis allowed us to identify refractory and D2T-RA patients and profile their characteristics. It was previously demonstrated that diverse patient-related and -nonrelated factors associated with D2T-RA, such as limited drug options due to contraindications and/or adverse events, comorbidities, the lack of concordance between the patient and the physician global assessment, and chronic pain conditions such as osteoarthritis and fibromyalgia [11, 15, 16]. Data from our population demonstrated that among refractory patients, D2T-RA are often male, younger, and with longstanding disease. Moreover, the analysis of pD2T-RA highlighted that this subgroup of patients previously failed a higher number of b/tsDMARDs and made more use of painkillers, although the rate of coexisting osteoarthritis and fibromyalgia did not diverge compared to eD2T-RA. However, multivariate analysis confirmed a significant association with higher SDAI scores, CRP values, and use of glucocorticoids (noteworthy, all definition criteria), confirming a more frequent use of analgesics as associated with pD2T-RA patients. These data look reassuring as the main pD2T-RA features observed in our real-life cohort appear to be concordant with the EULAR D2T-RA definition. Nevertheless, as the focus of our study was to identify patients with a persistent condition of difficult management, it appears that a concerning gaze should shift upon those patients that we categorized as episodic D2T-RA patients. With continuous, temporary fluctuations in disease activity state, our real-life cohort showed that up to one-third of D2T-RA patients possibly enrolled at any moment is constituted of patients who would not be categorized as such just a couple of months before and would not fulfill the D2T-RA definition a few weeks later. To avoid possible future errors in D2T-RA patients’ gatherings, we think that adding a temporal-related criteria of persistence would help to enroll more homogeneous difficult-to-treat populations.

Ultimately, the analysis of the rate of criteria fulfillment showed that the moderate disease activity criterion, the CRP criterion, and the glucocorticoids criterion were all significantly more frequently met among pD2T-RA patients rather than eD2T-RA. The ANOVA and Tukey tests did not reveal any significant differences in their fulfillment within the pD2T-RA population, even if a trend for a more frequent inability to taper glucocorticoids emerged. Noteworthy, all patients with symptoms of impaired quality of life were represented by patients with at least a moderate disease activity at enrollment. This finding is likely explained by the difficulty of retrospectively retrieving an objective definition of impaired quality of life from clinical records other than by an increased pain score.

Altogether, these results seem to support ideas and observations revolving around the concept of D2T-RA patients, such as the number of previously withdrawn biological or targeted synthetic therapies as surrogates markers of complexity [16, 18] and the differentiation of heterogeneous subgroups of D2T-RA, where patients presenting with imaging signs of inflammation are recalled as “true refractory RA” or “persistent inflammatory refractory”, while the other patients’ complex management seem to rely on other, non-inflammatory pain mechanisms [12, 15]. In addition, the characterization of D2T-RA and pD2T-RA patients highlights the presence of traits likely present before reaching the refractory state and so, potentially modifiable. In particular, our data points to a more prolonged time to b/tsDMARDs therapy as a feature of persistent D2T-RA trait, and the intensity and efficacy of therapies during the first months of the disease history seem to prevail as a factor potentially leading to a more drug-resistant and complex disease course [17, 19].

In the process of defining the D2T-RA status duration, we selected an observation period of 12 months as we perceive this time frame to be sufficiently long to unveil true cases of persistent D2T-RA patients and only temporary D2T-RA cases, as well as sufficiently short to allow oscillations of one patient’s disease activity state on one end of the time frame to resonate in the perception of complex management. Nevertheless, a known limitation of this study is the time-related D2T-RA was arbitrarily decided based on our experience and retrospectively assessed, and thus it could not be fully shared.

Some limitations to our study need to be mentioned, the first and foremost of which is the monocentric nature of the research and thus the limited number of patients included in the study. Moreover, data regarding reasons for b/tsDMARDs (i.e. primary or secondary inefficacy, adverse events, or contraindications) was not available for a significant proportion of patients and is currently being recollected, so analysis on any differences in mechanisms of therapy withdrawal between persistent and non-persistent D2T-RA was not carried out for this study. For the same reason, the identification of “true persistent RA” or persistent inflammatory refractory RA (PIRRA) through the presence of persistent inflammatory (i.e. synovitis) at the ultrasound evaluation was available only for a small, not representative sample of the population.

Furthermore, no patients with symptoms related to impaired quality of life were identified in our study. However, this finding may be because the employment of a pain score as a proxy for the definition criteria (chosen because of the retrospective nature of data collection) is insufficient to objectively picture the extent of the prevalence of symptoms of impaired quality of life despite a well-controlled disease. Thus, further prospective works with the application of specific tools may uncover this gray area.

Lastly, some future perspectives arising from the results of our study should mentioned. As we observed that one-third of the patients are represented by episodic D2T-RA, further studies should aim at identifying if any pharmacological and non-pharmacological interventions are preferentially associated with an “escape” from the D2T-RA definition. To accomplish so, further, prospective studies with a proper stratification of patients regarding the presence or absence of residual inflammation should guide the next steps in this direction.

## Conclusions

Through the first time-related analysis of D2T-RA patients, we demonstrated that only a proportion of refractory patients present with a condition of persistent D2T-RA disease. This population subgroup could represent simultaneously a deeper layer of complexity in the basin of D2T-RA patients and a more homogeneous population for research purposes. We believe that further clinical and biological characterization of persistent D2T-RA patients could lead to the implementation of prevention and management strategies as well as to a deeper understanding of pathologic mechanisms underlying RA prognosis and response to treatments.

## Data Availability

The datasets used and/or analysed during the current study are available from the corresponding author on reasonable request.
